# The potential of COPCA's coaching for families with infants with special needs in low- and middle-income countries

**DOI:** 10.3389/fped.2023.983680

**Published:** 2023-03-30

**Authors:** Schirin Akhbari Ziegler, Rosane Luzia de Souza Morais, Lívia Magalhães, Mijna Hadders-Algra

**Affiliations:** ^1^ School of Health Sciences, Institute of Physiotherapy, Zurich University of Applied Sciences ZHAW, Winterthur, Switzerland; ^2^ Department of Physiotherapy, The Federal University of Vales do Jequitinhonha e Mucuri, Diamantina, Brazil; ^3^ Department of Occupational Therapy, Federal University of Minas Gerais, Belo Horizonte, Brazil; ^4^ University of Groningen, University Medical Center Groningen, Department of Pediatrics, Division of Developmental Neurology and University of Groningen, Faculty of Theology and Religious Studies, Groningen, Netherlands

**Keywords:** family-centered, early intervention, low-and middle-income countries, high risk infants, COPCA, coaching

## Abstract

Infants at high biological risk of or with a neurodevelopmental disorder run a high risk of delayed school readiness. This is especially true for infants in low- and middle-income countries (LMICs). This perspective paper first summarizes evidence on intervention elements that are effective in promoting family well-being and child development in infants at high biological risk in high income countries. Crucial elements are family centeredness, goal orientation, a home setting, focus on activity and participation, and challenging the infant to explore the world and the own body by means of self-produced movements. The studies revealed that coaching as applied in COPCA (COPing and CAring for infants with special needs) is a pivotal element determining the success of intervention.The paper continues by describing COPCA and its coaching. Next, we report on two pilot studies addressing COPCA's implementation in Brazil. Finally, we discuss why COPCA is a promising early intervention program for infants at high biological risk of neurodisability in LMICs: COPCA is adapted to the families' strengths and needs, it empowers families and promotes child development therewith facilitating school readiness. Moreover, it may be delivered by tele-coaching therewith eliminating families' burden to travel to distant intervention clinics.

## Introduction

Infants at high biologically high risk of neurodevelopmental disability are, for example, infants born preterm or infants with a neonatal hypoxic-ischemic encephalopathy. Neurodevelopmental disability consists of a heterogeneous group of disorders, including cerebral palsy (CP), intellectual disability and autism spectrum disorders (1). The disorders affect multiple domains of activities and participation, such as mobility, learning and applying knowledge and communication (1). The presence of neurodevelopmental disability puts children at risk of limited school readiness. This is true for children world-wide, but the problem of limited school readiness is particularly pressing in low-and middle-income countries (LIMCs), as a high proportion of children with neurodevelopmental disability live in these countries (2) where early and appropriate intervention support to children and families is less available due to financial issues or limited accessibility (3).

It is generally agreed that infants and children at high risk of or diagnosed with neurodevelopmental disability should receive early intervention (4, 5). Literature suggests that effective early intervention programs are family-centered, goal-oriented, occur in the home setting in an enriched environment, focus on activity and participation, and challenge the infant to explore the world and the own body by means of self-produced motor behavior with trial and error. In addition, constrained-induced movement therapy or bimanual training are recommended for infants with clear asymmetries or unilateral CP, and early provision of assistive devices is recommended in infants who in early life already show substantial mobility limitations, e.g., due to a brain lesion. It is gradually acknowledged that children benefit more from the implementation of development-enhancing strategies during daily activities than from intervention activities more or less restricted to the intervention sessions themselves, as the child has more opportunities to practice in the former than in the latter situation. Coaching of the family members is a successful and modern means to let families appreciate how they in their own way can promote their child's development (1, 4).

Coaching is increasingly applied in early intervention and pediatric rehabilitation to foster family empowerment and child development. However, the application of coaching approaches confronts health professionals with challenges involving changes in professional role and associated behavior, and acquisition of coaching skills (6). Examples of coaching approaches designed for this field, with growing evidence for the effectiveness of coaching are “Coping with and caring for infants with special needs” (COPCA) (7, 8), Occupational Performance Coaching (OPC) (9), and Solution-Focused Coaching in Pediatric Rehabilitation (SFC-peds) (10). In COPCA positive associations between coaching of family members and (a) infant mobility and (b) empowerment and quality of life of the family have been demonstrated (11–14). OPC has been associated with positive effects on parents' self-efficacy and self-competences and on participation and occupational performance of children with neurodisability (15). Other studies suggest that SFC-peds is beneficial for the attainment of participation and friendship goals and increased sense of empowerment of children and youth with disabilities, and for the enhancement of skills and knowledge of their parents. All these approaches are family-centered and use reflection and feedback as intervention strategies (16–18). COPCA does not use video-feedback to coach families. Video-feedback is, for instance, used in situations in which families have established already problematic interactions with their children (19). In these situations, video-feedback helps family members to discover and correct maladaptive behavior. In COPCA, i.e., in the situation of intervention in early childhood the situation is different, maladaptive interactions did not have time to develop. COPCA's goal is to enhance the families' own capacities to solve problems. To this end, it uses dialogue with the family, shared observation of daily care giving activities (without video), and provision of hints and suggestions.

Coaching is a major ingredient of the early intervention program COPCA. In the following sections of this perspective paper, we first describe COPCA and the characteristics of COPCA's coaching. In the next section we report on two pilot studies addressing COPCA's implementation in Brazil. In the last section we discuss why COPCA, and its coaching strategy turns COPCA into a promising early intervention program for infants at high biological risk of neurodisability in LMICs: COPCA is adapted to the family's strengths, needs and culture, it empowers the family, it promotes child development and—ultimately—this will result in increased school readiness. Moreover, COPCA may be delivered by tele-coaching therewith eliminating the family's burden to travel to distant intervention clinics.

## COPCA and coaching in COPCA

COPCA is a family-centered early intervention program, which includes all above mentioned components (7, 8). Becoming a COPCA coach requires a professional education course of 3 × 2 days and two individual coaching sessions of one hour (8). COPCA has been designed for infants at high biological risk of neurodisability. COPCA has two aims: 1) to enhance empowerment of individual families in the process of decision-making regarding activities and participation of child and family; and 2) to promote infant development in general and especially the child's mobility allowing for optimal participation in daily life and to prevent contractures and deformities.

Coaching is COPCA's major strategy. The goal of coaching is to empower family members to discover their own strategies, capacities, and competences to challenge the infant with special needs in naturally occurring parenting situations. COPCA's coaching approach is goal-oriented and complies with the three criteria of Ives (20): it is non-directive, solution-focused and performance driven. Being non-directive implies that the coach is a facilitator and stimulator of ideas and actions and not a trainer or instructor. Solution-focused implies a focus on finding solutions to achieve specific aims. Being performance driven emphasizes the focus on changing actions to improve performance through understanding of circumstances.

In COPCA family members are equal and active partners in the intervention. They are actively involved goal setters, decision makers and supporters of the child with special needs. They are engaged in daily care activities in naturally occurring parenting situations. In COPCA health professionals act as a coach. In this role health professionals observe, listen, ask, and provide information. The coach honors families as experts of their lives and believes that every family member is creative and resourceful. In coaching, relationships between family members and health professionals are of critical importance.

COPCA coaches focus on the whole family as a unit, implement equal partnership and recommend families to find their own solution. Therefore, COPCA coaches respect families' autonomy and acknowledge families' own criteria for quality of life. The coach has confidence in families' competences and capacities: family members are the key persons in the intervention. The family's values, routines and rituals are respected. COPCA takes place in an enriched real-life environment, during daily care giving activities like playing, dressing, feeding, or bathing. Enriched implies that caregivers receive hints and suggestions how they can use material available in the home environment to play with the child. No expensive material is needed. In COPCA sessions, family members receive coaching on how to promote infant development, for instance how to challenge the infant to self-produced motor behavior. This involves discussions of coach and family members on how to offer the infant opportunities to explore the environment, and how to let the infant experience trial and error. To this end the coaching strategies specified in Table 1 are used. COPCA coaches appreciate the unique situation of each family, including the family's cultural background. They recognize the families' coping strategies and offer tailored interventions that are adapted to the strengths, resources, decisions, goals and needs of the family members and the child with special needs. The coaching strategies are adapted to the individual needs of the family in a non-directive way. Typically, the COPCA intervention starts with the COPCA coach visiting the family once a week for 45 to 60 min. After a few weeks, the frequency can usually be reduced to every two weeks and later once a month. Since the intervention is adapted to the individual needs of the family, the procedure is flexible.

**Table 1:** Coaching strategies in COPCA.

Coaching Strategies	Definition
Information exchange	Information exchange means all communication tuned to the guidance of the infant and the family as an entity. This includes exchange of knowledge, and exchange of information related to the development of the infant or the actual situation of the infant and family.
Active listening	Active listening implies to listen attentively and concentrated. Paying attention to nonverbal signs of the partner, and—when needed—respond to the non-verbal signs.
Shared observation	Shared observation means that the caregiver and health professional jointly observe the infant's motor activities, or that the health professional observes caregiver-infant interactions during daily activities, and that caregiver and health professional share their observations with each other.
Provision of hints and suggestions	Hints and suggestions invite caregivers to implement their own strategies aiming to promote child development or to evolve own ideas during the implementation.
Asking reflective questions	Reflection means scrutinizing and comparative mediation about different aspects of knowledge, skills, desires, aims, actions, or observations. It includes the evaluation of behaviors and/or results of the current intervention. Reflection enables realization, analyses and/or generation of alternative behavior strategies to better reach the own aims. Questions which may inspire reflection, are called reflective feedback.
Provision of feedback	Two different kinds of feedback may be provided, informative feedback and affirmative feedback. Informative feedback means to share information directly related to an action of the caregiver or to an observation. Affirmative feedback means to affirm an action or information of the caregiver.
Illustration with example	The health professional explicitly models an intervention strategy (with the infant or a doll) and the caregiver observes the action of the health professional or acts together with the health professional. The example serves as a hint or suggestion, it does not aim at instruction. Typically, the illustration is accompanied by verbal information and followed by actions of the caregiver with the infant.
Joint planning	Joint planning means that at the end of the session, the parents and the coach together plan which activities the family will try out during daily routine activities during the interval between the sessions.

The effectiveness of COPCA's coaching strategies in infants at high biological risk of neurodevelopmental disorders has been demonstrated in three randomized controlled trials performed in high-income countries (HICs). Coaching of family members was not only associated with improved cognition and better mobility of the infant in daily life, but also with better family empowerment and well-being (11–14, 21). Interestingly, the positive effect of COPCA intervention continued after the end of the intervention (measured about one year after the randomized interventions had stopped), suggesting that families had learned the principles of COPCA on how to stimulate their child's development during daily life activities (11, 14).

## Benefit of COPCA'S coaching in LMICs: The example of Brazil

### Pediatric health and developmental care in Brazil

Brazil is a country with a large territorial extension and cultural diversity. Although access to remote areas and wealth inequalities challenge universal coverage by public health services, child health and nutrition indicators improved considerably over the last three decades (22). Nonetheless, problems ranging from absence of universal coverage of basic sanitation to limited access to health care services persist. Brazil belongs to the ten countries with the highest rate of preterm births worldwide (11.2% of live births) (23). As in other LMICs, it is common that children pair biological risk with psychosocial risk, resulting in risk accumulation (24). Psychosocial risk factors include food restriction, low parental education, and poor social and environmental stimulation.

Only a few studies addressed the prevalence of developmental delay in Brazil. A population-based study, performed in the Northeast, revealed that 9.2% of children 0–6 years were delayed in at least one developmental domain (25). Another study from the Northeast in infants aged 0–28 months reported that 23% of infants were suspected of a delay in personal-social skills and 20% of a delay in language skills (26). A third study, carried out in Brazil's south, indicated that 32% of infants aged 0–36 months were suspected of developmental delay (27). We also know that the prevalence of disabilities among children aged under 5 years in Latin America and the Caribbean is higher than that in Europe, Central Asia and North America, but not as high as in South Asia and Africa (2). These data—varied as they may be—underline that a considerable proportion of young Brazilian children need early intervention services.

Access to early intervention in Brazil is, however, not easy. It is linked to access to health care in general, which occurs either through the private system—for the minority of the population that can afford it—or through public health care, i.e., through the Unified Health System (Sistema Único de Saúde (SUS)). The SUS, which is used by most people, offers full, universal, and free access to health services. It has front doors at the community level, with basic healthcare units for primary care. Implemented in 2015, the Brazilian National Policy on Integral Attention to the Health of the Child (28) prioritizes health care actions for young children, especially for those in a vulnerable context. The actions start with humanized and qualified care during pregnancy, childbirth, and care for the newborn, including actions directed to preterm and low birth weight newborns offered by SUS. The latter care ranges from kangaroo care and specialized hospital care to shared infant follow-up by hospitals, university centers and primary care teams. Infants, who during follow-up are diagnosed with a delayed or atypical development, are referred for early intervention to rehabilitation or specialized centers, such as the Associação de Pais e Amigos dos Excepcionais (29).

### COPCA in Brazil

A recent review on early intervention (30), indicated that in Brazil a rehabilitative model of care is used, i.e., a model applying clinical approaches and child-centered care. This means that early intervention in Brazil in general differs from the internationally recommended good practices (5). Therefore, we recently embarked on the implementation of COPCA in Brazil, as COPCA has several advantages to the typical early intervention approaches in Brazil. First, COPCA fully complies with the international guidelines. Second, COPCA is family centered and home based. This means that family members are coached to find their own ways in rearing the child with special needs in their own home environment. This also implies that families do not need to travel to a center that provides early intervention, and that, ultimately, less health professionals are needed. The latter is an advantage in a country with overall shortage of health professionals (31), including those specialized in early intervention.

A recent Brazilian law, that provides guidelines to formulate and implement public policies for young children (32), and the national healthcare policy (20) recommend early intervention at home. Nonetheless, only a few of such programs are currently available, for example “Primeira Infância Melhor” (22) and “Programa Criança Feliz” (33). These programs are, however, aimed at children at psychosocial risk, not at children at high biological risk. A study from Ghana (Fonzi et al., 2021) indicated that also caregivers of children with cerebral palsy preferred to receive home care, as home care was associated with a reduction of treatment costs, caregiver burden and social stigma (34). Intervention at home with possible cost reduction may be strategic, as some families have difficulties to attend follow-up sessions in clinics due to economic challenges (35).

The use of COPCA's coaching techniques ensures that families are supported in advancing problem-solving strategies to promote development of their child with or at high risk of neurodevelopmental disabilities. COPCA also provides families with opportunities to learn about child development in the context that makes sense to the family. Last, but not least, COPCA's coaching results in family empowerment and may help families to be more assertive throughout a lifetime of care. Current recommendations for improving health and social systems for children in LMICs indeed include redesigning health service delivery models to maximize outcomes, not only to empower families to better care for children, but also to demand better services (36).

**Figure 1:**
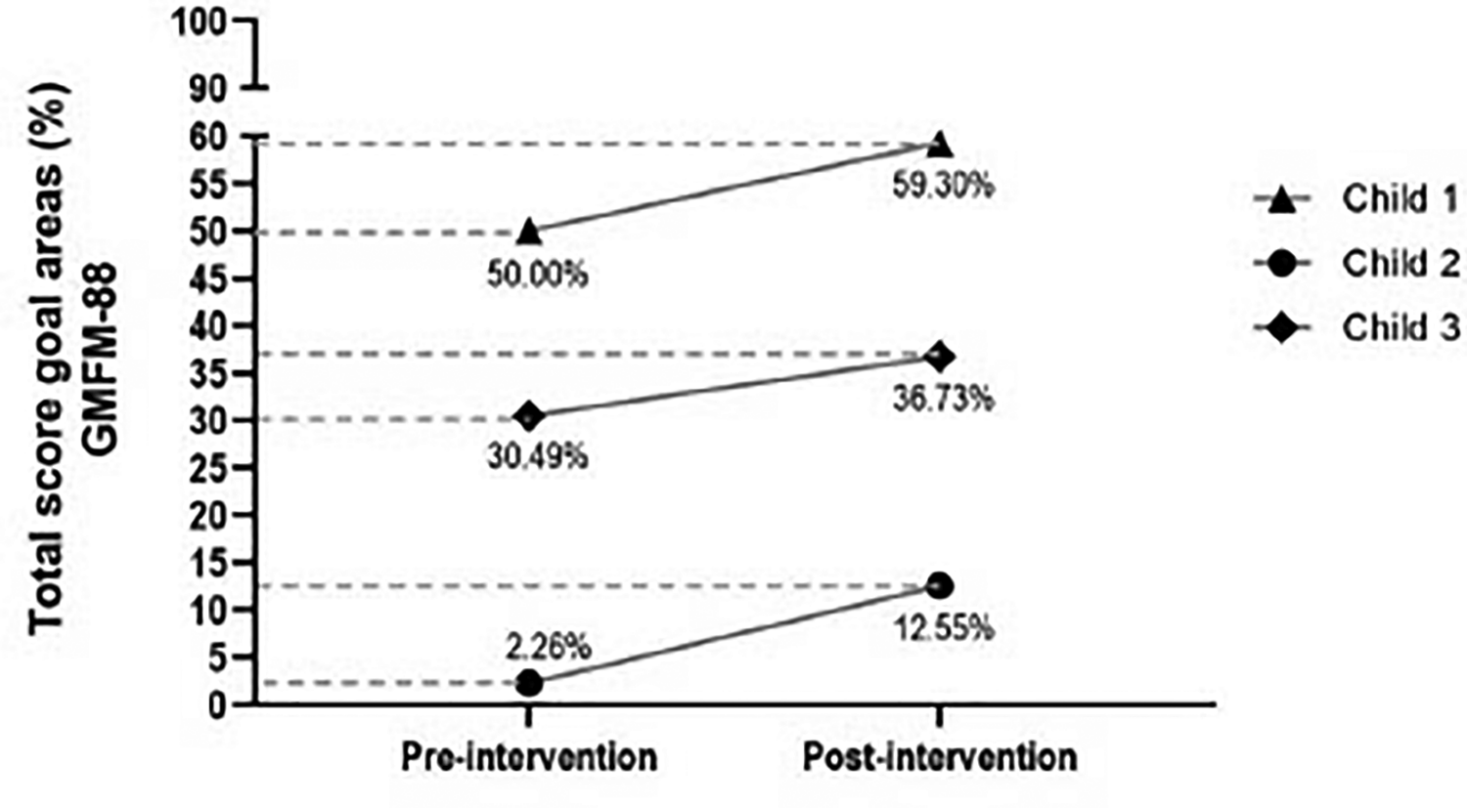
Changes in GMFM-88 percentage scores in the goal areas of the three children with CP of the Brazilian case series study during the 7 weeks of Family-Centered Care intervention (led by a COPCA coach). Figure reprinted from reference 38 with permission of Revista Fisioterapia em Movimento (Curibita, PR, Brazil: DOAJ).

We recently reported about our experience with COPCA in Brazil (37) in a case series study with five Brazilian children. Four of the five families had a low income. Three children had been diagnosed with cerebral palsy (Gross Motor Function Classification System levels III, IV and V), one was an infant at high biological risk due to perinatal hypoxia/ischemia, and another child had psychosocial risk due to adverse childhood experiences. The children's families were coached by physical therapy students, who were supervised by a certified COPCA® coach. The families received seven weekly one-hour home visits, with COPCA coaching. After the seven weeks of intervention, the three children with cerebral palsy showed an increase of more than 5% in the target areas of the Gross Motor Function Measure (GMFM-88) (38), a gain that is considered clinically important (Figure 1). In addition, the percentile ranking score on the Albert Infant Motor Scale (AIMS) (39) increased in the infant at biological risk. The AIMS percentile scores of the infant at psychosocial risk did not change during the intervention (Table 2). The latter may have been due to the student's limited experience to cope with the challenging psychosocial needs of the family. The study also showed that all families were very satisfied with the results obtained during the short intervention—also the family of the infant at psychosocial risk—and their responses indicated that they felt empowered.

**Table 2:** Changes in the Alberta infant motor scales (AIMS) scores in the two children without CP of the Brazilian case-series study.

	Child 4		Child 5	
	Pre-intervention	Post-intervention	Pre-intervention	Post-intervention
Percentile	**<25**	**50**	**10**	**10**
Total score	**15**	**29**	**49**	**52**
Prone	4	8	21	21
Supine	6	8	9	9
Sitting	3	10	12	12
Standing	2	3	7	10

Percentile scores based on Piper & Darrah, 1994 (39). Table adapted from Cunha et al. (37). Child 4 was at high biological risk due to perinatal hypoxia/ischemia, child 5 was at psychosocial risk due to adverse childhood experiences.

**Figure 2:**
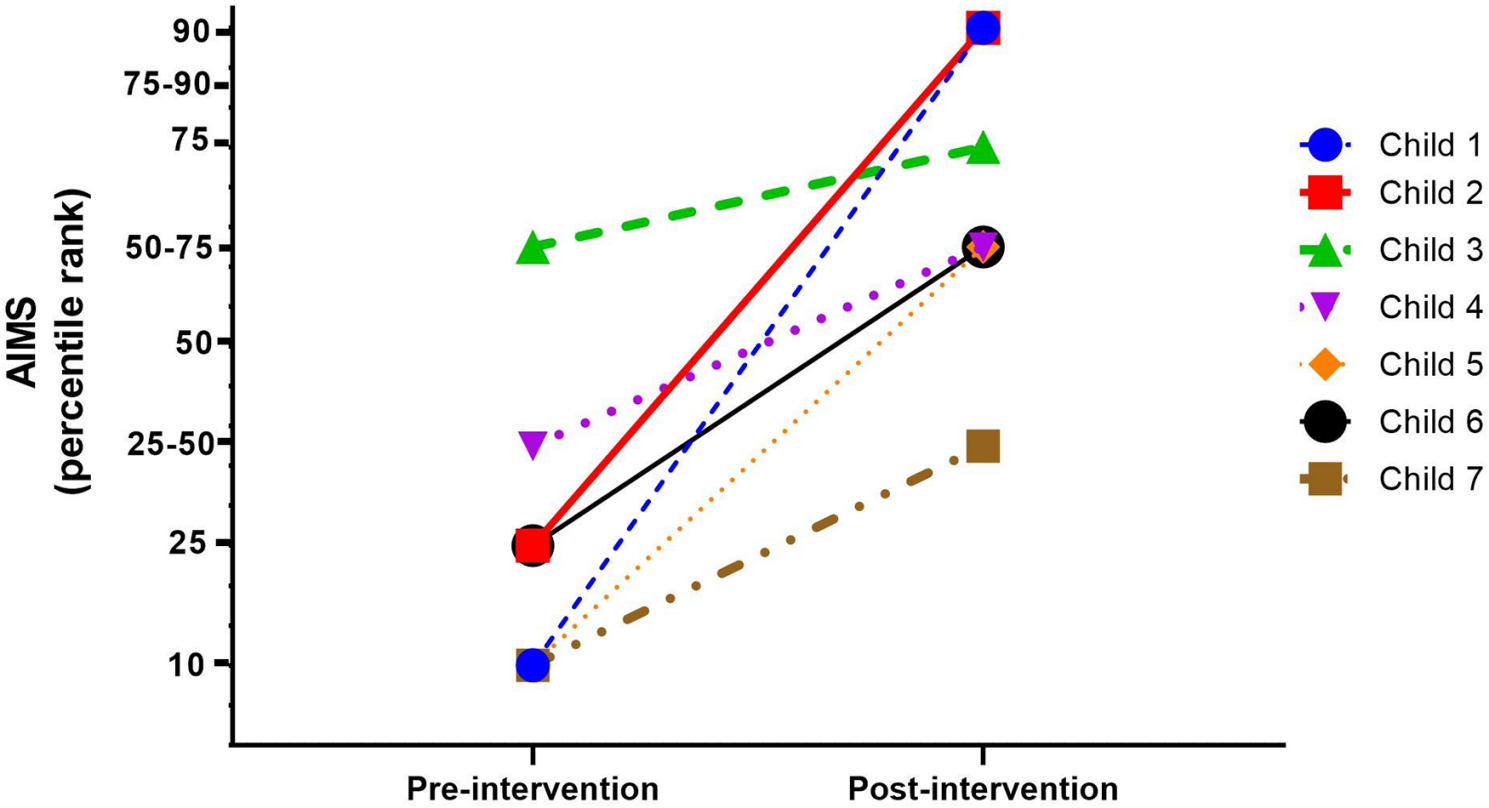
Developmental trajectories in percentile rank scores of the AIMS of the seven preterm infants before and after 8 weeks of COPCA intervention (results of the second Brazilian pilot study).

In another study, performed during the COVID pandemic, physical therapy students provided intervention supervised by a certified COPCA® coach in seven preterm infants (gestational age at birth 29–36 weeks; correct age at start intervention 5–14 months corrected ag) *via* telemonitoring. This means that we implemented COPCA's coaching *via* the video-call option of WhatsApp. After eight weeks with a weekly tele-COPCA-coaching session all infants had reached the goal that had been determined in partnership with the families at the beginning of the intervention. In addition, all infants showed a substantial increase in the AIMS percentile scores (Figure 2). Moreover, the caregivers were satisfied with the results and felt supported and empowered by the approach.

The second case series showed that it is feasible to implement COPCA by means of tele-intervention. Tele-coaching of COPCA may be an attractive early intervention strategy for Brazil, as it eliminates the family's burden to travel to a clinic. The travel burden is a well-known factor reducing adherence to early intervention (40, 41). Tele-coaching of COPCA also enables a virtual visit to the infant's home. It therewith allows for the visualization of the natural home environment, and it facilitates exchange of information and discussion of activities that fit within the infant´s reality. In addition, as tele-guidance makes it impossible for the health professional to touch and handle the child, the transition to a really family-centered approach is more easily achieved. In other words, tele-guidance facilitates the implementation of COPCA's coaching strategies. Tele-guidance can also be used in combination with face-to-face care; for instance, in families who live in distant communities or in rural areas, which is very common in Brazil.

Conceivably, one barrier to the implementation of COPCA, not investigated in these two case studies, maybe parental objection to the novel approach of coaching, as it so different from the most used approaches to developmental physical therapy in Brazil. These interventions consist of hands-on approaches such as neurodevelopmental treatment and suit therapy (42). In these traditional approaches, the therapist acts as an expert, who handles the child and instructs parents what to do. COPCA's coaching implies a different role of the family members, which might meet resistance. However, it should be realized that in the countries in which COPCA was first applied, i.e., in the Netherlands and Switzerland, similar primary worries on COPCA's implementation existed. Nonetheless, COPCA's implementation in daily practice revealed that the families gladly accepted the new approach, after having received information of the approach's background. In the Brazilian case studies the parents in the study seemed to appreciate COPCA, but it must be noted that parents might have been motivated to try COPCA as some children had not been making gains with the traditional approaches, and the other children were either on a waiting list or did not have access to other forms of intervention due to the COVID-19 pandemic. This means that future studies need to address in the LMICs the perception of family members of COPCA, including its advantages and disadvantages compared to traditional approaches.

Based on the theoretical reflections, the overall child health care situation in Brazil, and the promising results of the pilot studies, we believe that COPCA is an early intervention program that may contribute to overcoming the challenges encountered in Brazil's early intervention services for infants at high biological risk. Even though more data are needed to support this assertion, there were no barriers to the application of COPCA coaching with low-income families. COPCA fits to Brazil's primary care because its coaching strategy works with the resources that are available in the home environment, and the family does not need to go to a rehabilitation center or pay for expensive equipment. COPCA's coaching may be delivered face-to-face or *via* tele-guidance or by a combination of both approaches.

## Discussion and conclusion

Between 1990 and 2020 mortality in children aged under 5 years decreased by 60% due to the impact of the United Nations' Millennium Development Goals (3). Fortunate as this may be, this gave—in combination with the rapid population growth in LMICs—also rise to an increase of infants at high biological risk of neurodevelopmental disorders. As a result, more than 90% of children with disabilities live in LMICs (3, 43). This implies that the need of adequate early intervention in LMICs is high.

Most early intervention programs in LMICs focus on families in challenging social conditions, for instance families dealing with poverty (44). Early intervention in these situations is most effective when it consists of parenting interventions, i.e., of intervention that aim to improve caregivers' knowledge, attitudes, practices, and skills, including responsive caregiving. Such interventions allow caregivers to promote in their own situation optimal early child development (18, 45).

Little is known on early intervention in the infants with highest needs, i.e., the infants at high biological risk of neurodisability in LMICs (46, 47). It may be assumed that they will benefit from the same intervention strategies that are profitable for infants at high biological risk in HICs. But knowing which interventional elements are effective in promoting developmental outcome is one thing, implementing early intervention in challenging social situations, as frequently met in LMICs, is quite another thing. The intervention needs to reach the families (48). The latter implies that the intervention has to take into account the families' culture, perceptions, finances and levels of stress (48).

Our preliminary data with COPCA in Brazil suggest that COPCA is an early intervention program that may serve early intervention in infants at high biological risk in LMICs. COPCA's coaching strategies are tailored to the needs of individual families, as family autonomy is a crucial element in COPCA. Family members learn through the empowering dialogue with the COPCA coach in which way they can promote their child's development, in their own situation according to their own cultural norms. In addition, COPCA's coaching may be delivered by tele-coaching therewith eliminating the family's burden for travelling to distant early intervention clinics. Larger scale studies are needed to support COPCÁs effectiveness for early intervention in LMICs as well as to identify possible barriers to its implementation and how to overcome them. Intervention by health professionals is associated with substantial costs, which may hamper the implementation of the intervention. We also recommend studies that evaluate which part of the COPCA intervention may be delivered by lay or paraprofessional community health workers and which part needs to be taken care of by fully educated COPCA coaches.

In conclusion, LMICs face the challenge of implementation of effective early intervention services for a high number of infants at high biological risk of neurodisability. Increasing evidence in HICs indicates that interventions in which families are empowered to find their own solutions, on how they can promote their child's development during daily care giving activities, are associated with better child development and favorable family outcome. A major strategy to reach these goals is coaching, which is COPCA's fundamental intervention strategy. Two pilot studies in Brazil indicated that COPCA's coaching technique, including its tele-coaching option, turns COPCA in a promising early intervention for infants at high biological risk in LMICs. COPCA's positive effect on family empowerment and child development suggest that COPCA may be associated also with improved school readiness.

## Data Availability

Publicly available datasets were analyzed in this study. This data can be found here: Cunha RFM da, Costa KB, Morais RL de S. Family-centered care on a physiotherapy course: case reports. Fisioter mov (2022) 35: doi: 10.1590/fm.2022.35301.
